# A self-amplifying RNA vaccine provides protection in a murine model of bubonic plague

**DOI:** 10.3389/fmicb.2023.1247041

**Published:** 2023-11-02

**Authors:** Robin John Shattock, Voahangy Andrianaivoarimanana, Paul F. McKay, Lovasoa Nomena Randriantseheno, Valarmathy Murugaiah, K. Samnuan, Paul Rogers, John S. Tregoning, Minoarisoa Rajerison, Kristoffer M. Moore, Thomas Robert Laws, E. Diane Williamson

**Affiliations:** ^1^Dept. of Infectious Disease, Imperial College London, London, United Kingdom; ^2^Plague Unit, Institut Pasteur de Madagascar, Antananarivo, Madagascar; ^3^CBR Division, Dstl Porton Down, Salisbury, United Kingdom

**Keywords:** saRNA, mRNA, vaccine, plague, efficacy, mouse, bubonic plague, *Yersinia pestis*

## Abstract

Mice were immunized with a combination of self-amplifying (sa) RNA constructs for the F1 and V antigens of *Yersinia pestis* at a dose level of 1 μg or 5 μg or with the respective protein sub-units as a reference vaccine. The immunization of outbred OF1 mice on day 0 and day 28 with the lowest dose used (1 μg) of each of the saRNA constructs in lipid nanoparticles protected 5/7 mice against subsequent sub-cutaneous challenge on day 56 with 180 cfu (2.8 MLD) of a 2021 clinical isolate of *Y. pestis* termed 10-21/S whilst 5/7 mice were protected against 1800cfu (28MLD) of the same bacteria on day 56. By comparison, only 1/8 or 1/7 negative control mice immunized with 10 μg of irrelevant haemagglutin RNA in lipid nanoparticles (LNP) survived the challenge with 2.8 MLD or 28 MLD *Y. pestis* 10-21/S, respectively. BALB/c mice were also immunized with the same saRNA constructs and responded with the secretion of specific IgG to F1 and V, neutralizing antibodies for the V antigen and developed a recall response to both F1 and V. These data represent the first report of an RNA vaccine approach using self-amplifying technology and encoding both of the essential virulence antigens, providing efficacy against *Y. pestis*. This saRNA vaccine for plague has the potential for further development, particularly since its amplifying nature can induce immunity with less boosting. It is also amenable to rapid manufacture with simpler downstream processing than protein sub-units, enabling rapid deployment and surge manufacture during disease outbreaks.

## Introduction

Currently, there is no approved and accessible vaccine for plague, which remains a life-threatening endemic disease in global regions (WHO R&D blueprint, 2018). In recent years, significant advances toward an approved vaccine have been made (WHO R&D blueprint, 2018), including the exploitation of immunogens and further characterization of the key protective antigens, F1 and V. When secreted by the causative bacterium *Yersinia pestis* during infection, these antigens control important virulence mechanisms: F1 is exported to the bacterial surface where it is the major component of a protective capsule that has anti-phagocytic activity (Friedlander et al., 1995; Tito et al., 2001); the V antigen is key to the bacterial Type 3 secretion mechanism during which, on host cell contact, an injectisome is formed through which V antigen is exported and reassembled on the injectisome’s tip where, with the aid of pore-forming Yersinia outer proteins, it is delivered to the host cell with cytotoxic and immunomodulatory effects (Mueller et al., 2005; Cornelis, 2006).

These sub-units have been produced in a recombinant form, either as a fusion protein (F1-V) or in simple admixture (F1 + V), and have been shown to be highly immunogenic and efficacious to protect a range of animal models against either injected or aerosolised exposure to *Y. pestis* (Sun and Singh, 2019). Additionally, both presentations of the protein sub-units (F1-V and F1 + V) have been evaluated in clinical trials and have been shown to be safe and immunogenic in many hundreds of volunteers (Williamson et al., 2005; Hart et al., 2012).

However, the scaled-up expression and GMP manufacture of these protein sub-units and their subsequent formulation is an expensive and labor-intensive exercise that does not lend itself well to an emergency requirement. Messenger RNA (mRNA) vaccine manufacture offers a more streamlined approach to translational development since mRNA constructs for F1 and V can be prepared and formulated at GMP and deployed directly. As a further modification, by incorporating a viral replicase in the RNA constructs these become self-amplifying (sa) *in vivo* (McKay et al., 2020), providing a potential lower-dose and cost-effective alternative to mRNA. In this context, the use of lower doses maximizes the number of doses per production batch, which is also advantageous in outbreak situations (Kis et al., 2020).

Here, we prepared saRNA constructs for the two key protective antigens, F1 and V. We immunized both inbred and outbred mice with these combined constructs, which were formulated and delivered intra-muscularly (i.m.) in lipid nanoparticles (LNP), and we assessed the immune responses induced. Subsequently, we challenged the mice by the sub-cutaneous (s.c.) route with a recent Malagasy clinical isolate of *Y. pestis* (*Y. pestis* 10-21/S) (Andrianaivoarimanana et al., 2021) to determine the protective efficacy induced.

These data represent a significant step forward in the search for an easily manufactured and more broadly protective vaccine for plague and build on recent data from Kon et al., who showed that an RNA construct encoding only F1 was efficacious in mice against the virulent KIM53 strain of *Y. pestis* (Kon et al., 2023).

## Materials and methods

### Preparation of constructs for immunogenicity studies

In initial studies, individual DNA constructs for each F1 (*n* = 5) and V (*n* = 4) were prepared to determine the most immunogenic sequence on which to base subsequent RNA constructs. For an initial immunogenicity study, these DNA constructs were administered i.m. by electroporation (Mann et al., 2014). DNA constructs were designed as follows: V1 = secreted monomer, where a secretion signal peptide was placed upstream of the original bacterial sequence; V2 = truncated secreted monomer (V1), with C-terminal truncation following N281 to improve potential secretion; V3 = membrane-tethered trimer, where the secreted monomer (V1) was fused to the D8 transmembrane domain of OPG105 from vaccinia to promote trimerisation; V4 = secreted V1-ferritin fusion protein where the secreted monomer of V1 was fused via a flexible linker (GSGGG) to a ferritin monomer, designed to promote the secretion of a self-assembling ferritin particle with V1 on the outer facing surface; F1 = the original bacterial F1 sequence with a deletion of the N terminal 21 amino-acid signal sequence; F2 = F1 with a 35 amino acid N-terminal deletion (ΔN) to remove additional hydrophobic residues with the aim of improving secretion; F3 = where a secretion signal peptide was placed upstream of the ΔN terminal sequence; F4 = where the transmembrane sequence of CD74 followed by a short linker was placed upstream of the ΔN sequence to provide a membrane tethered version with potential for cell surface display of the antigen; F5 = secreted F1-ferritin fusion protein where the F3 version of F was fused by a flexible linker (GSGGG) to a ferritin monomer to promote secretion of a self-assembling ferritin particle with F on the outer facing surface (see Supplementary Information for sequence data).

Each DNA open reading frame was inserted into a standard plasmid vector immediately after a CMV promoter for constitutive intracellular transcription, together with a Kozak translation initiation sequence, which was placed adjoining the ATG start codon. For the saRNA template plasmid constructs that were used to generate saRNA by *in vitro* transcription (IVT), the V or F1 sequences (selected from the options above) were inserted downstream of the viral replicase and subgenomic promotor of the Venezuelan Equine Encephalitis virus (VEEV), replacing the viral non-structural proteins, and assembled in a T7 expression plasmid as previously described (McKay et al., 2020). saRNA was generated by IVT with the final concentrations each of 5 mM NTP, 40 mM Tris–HCL pH 8, 10 mM DTT, 2 mM Spermidine, 0.002% Triton, 27 mM Magnesium Acetate, 20 U T7 RNA polymerase per 1 ug DNA template, 1 U/uL RNase inhibitor, and 5 mM CleanCap AU (N-7114, Trilink Biotechnologies, CA, USA). SaRNA was then formulated in a lipid nanoparticle (LNP), utilizing C12-200 (Corden Pharma, Switzerland) as the complexing ionizable lipid (N/P ratio of 8), 1,2-distearoyl-sn-glycero-3-phosphocholine (DSPC) (Avanti® Polar Lipids, US) as the helper lipid, cholesterol (plant-derived) (Avanti® Polar Lipids, US), and 1,2-dimyristoyl-sn-glycero-3-phosphoethanolamine-N-[methoxy(polyethylene glycol)-2000] (ammonium salt) (DMPE-PEG2000) (Avanti® Polar Lipids, US) as previously described (McKay et al., 2020). Z-average diameter and zeta potential were analyzed using a Zetasizer Nano ZS (Malvern Instruments, UK) and encapsulation using a Quant-iT RiboGreen assay (Thermo Fisher Scientific, UK). The Z-average diameter was 80–100 nm with a PDI of 0.2–0.3 and a Zeta potential of −4 to −6 mV. Encapsulation efficiency was >90%. The LNP-formulated saRNA vaccines were shipped on dry ice and stored at <-60°C before use.

### Immunogenicity testing

All animal studies were carried out according to protocols approved by the Institutional Animal Care and Use Committees in each location, at Imperial College London (ICL) and Institut Pasteur Madagascar (IPM). BALB/c mice were obtained from Charles River, and OF1 mice were bred at IPM.

In an initial screening study at ICL, the DNA constructs for F1 and V (10 μg each) were delivered intra-muscularly (i.m.) in the quadriceps with electroporation (Mann et al., 2014) to immunize female BALB/c mice (groups of 5) on days 0 and 28. On each occasion, post-injection, the sites received electroporation via tweezer electrodes from an ECM830 square wave EP system (BTX). Pulses were 100 V of positive or negative polarity at 1 pulse/s, with each lasting 50 mseconds.

Subsequently, a single saRNA construct for each of F1 [based on DNA construct F1 (1)] and V (based on DNA construct V1) was prepared for a further immunogenicity study at ICL, followed by immunogenicity and efficacy testing at the Institut Pasteur de Madagascar (IPM). At ICL, saRNA constructs for the down-selected F1 and V sequences were used to immunize 8 BALB/c mice at two doses (1 μg and 5 μg), i.m. At IPM, these saRNA constructs were used to immunize groups of 8 female outbred (OF1) mice using the same dose levels and route.

In each location, the immunization schedule was the same. On the day of each vaccination (0, 28), the saRNA vaccines were thawed at ambient temperature, mixed thoroughly, and used within 2 h. Mice were immunized i.m. in a total volume of 0.05 mL/mouse into the gastrocnemius muscle such that the F1 (1) construct was delivered into one hind leg in 0.05 mL i.m. and the V construct into the contralateral leg (0.05 mL i.m.). At ICL, mice underwent venepuncture on days 0, 21, and 35. At IPM, whole blood samples were collected and placed onto a seropad on days 0, 23, and 51 and then analyzed as previously described (Dromigny et al., 1998; Andrianaivoarimanana et al., 2021). The antibody response was determined by standard ELISA (Williamson et al., 2005), and the neutralizing antibody (NAb) response directed toward the V antigen was detected by competitive ELISA (Moore et al., 2022). The assay was conducted on murine test serum and pooled by the treatment group. NAb was assayed by competing the test serum for binding to the V antigen on the solid phase with the neutralizing biotinylated monoclonal antibody 7.3 (Mab 7.3). The binding of biotinylated Mab 7.3 was detected with a streptavidin peroxidase antibody, so a reduction in the OD signal indicated a reduction in biotinylated Mab 7.3 binding to the V antigen.

As previously described, splenocytes collected at day 35 were assayed by ELIspot (Tregoning et al., 2023). In brief, splenocytes (1.25 × 10^6^/ml) were restimulated *in vitro* with a combination of F1 and V antigens (20 μg/mL) and incubated overnight at 37°C, 5% CO2. IFNγ secretion was quantified by immunostaining, and the enumeration of the spots was read using AID ELISpot reader ELR03 with AID ELISpot READER software (Autoimmun Diagnostika GmbH, Germany). The data were reported as spot-forming units (SPU) per 10^6^ splenocytes.

### Efficacy testing

At IPM, the groups of female outbred OF1 mice (*n* = 8 per group) were allocated to treatments as shown in Table 1 and immunized i.m. as described above. Negative control groups received either 10 μg of irrelevant RNA (heamagglutinin, HA) encapsulated in LNP or 20% v/v alhydrogel, both administered i.m. For the reference vaccine, the F1 and V sub-unit proteins were combined, formulated in 20% v/v alhydrogel, and the vaccine was divided between each hind leg (0.05 mL/leg) to deliver 5 μg of each protein.

**Table 1:** Immunogenicity and efficacy testing in OF1 mice: treatment groups and schedule.

Group (*n* = 8)	Priming immunization day 0 (i.m.)	Booster immunization day 28 (i.m.)	Blood samples	Challenge with *Y. pestis* 10/21-S (s.cut) At 2 levels
1. RNA vaccine, 1 μg	RNA 1 μg F1 + RNA 1 μg V in LNP	RNA 1 μg F1 + RNA 1 μg V in LNP	days 0, 23 and 51	day 56 with 180 cfu
2. RNA vaccine, 5 μg	RNA 5 μg F1 + RNA 5 μg V in LNP	RNA 5 μg F1 + RNA 5 μg V in LNP	days 0, 23 and 51	day 56 with 180 cfu
3. Negative control	RNA 10 μg HA in LNP	RNA 10 μg HA in LNP	days 0, 23 and 51	day 56 with 180 cfu
4. RNA vaccine, 1 μg	RNA 1 μg F1 + RNA 1 μg V in LNP	RNA 1 μg F1 + RNA 1 μg V in LNP	days 0, 23 and 51	day 56 with 1800 cfu
5. RNA vaccine, 5 μg	RNA 5 μg F1 + RNA 5 μg V in LNP	RNA 5 μg F1 + RNA 5 μg V in LNP	days 0, 23 and 51	day 56 with 1800 cfu
6. Recombinant F1 and V vaccine, 5 μg	5 μg F1+ 5 μg V/20% alhydrogel	5 μg F1+ 5 μg V/20% alhydrogel	days 0, 23 and 51	day 56 with 1800 cfu
7. Negative control	RNA 10 μg HA in LNP	RNA 10 μg HA in LNP	days 0, d.23 and d.51	day 56 with 1800 cfu
8. Negative control	Alhydrogel	Alhydrogel	days 0, 23 and 51	day 56 with 1800 cfu

Mice were blood-sampled serially to monitor the immune response induced, and 28 days after the booster dose on day 56, they were exposed to *Y. pestis* 10-21/S (Andrianaivoarimanana et al., 2021) by the subcutaneous (s.c.) route at either a target dose of 10^2^ or 10^3^ cfu in 0.1 mL/mouse. The median lethal dose (MLD) of the capsulated *Y. pestis* 10-21/S isolate had previously been determined as 65 cfu (Andrianaivoarimanana et al., 2021). Prior to use, the *Y. pestis* 10-21/S isolate was grown in brain-heart infusion (BHI) broth (Oxoid Ltd., UK) for 2 days at 28°C to provide an inoculum and on Cefsulodin-Irgrasan-Novobiocin (CIN) agar plates to enumerate viable bacterial colonies.

IPM is a WHO Collaborating Center for Plague. The IPM facility for the safe handling of infected animals utilizes a biosafety cabinet with vertical laminar airflow venting to the atmosphere, and operators use personal protective clothing, gloves, and masks; this is classed as a P2+ operation, equivalent to BSL2 + .

Post-infection (p.i.), mice were closely monitored for 14 days, with those showing clinical signs representing the humane endpoint being culled by cervical dislocation. At 14 days p.i., surviving mice were also culled, and blood and spleens were collected post-mortem.

### Retrospective enumeration of challenge dose levels

The challenge inocula were retrospectively cultured on CIN agar plates (Rasoamanana et al., 1996) and enumerated to estimate the number of colony-forming units (cfu) delivered in a 0.1 mL inoculum per mouse.

### Bacteriology post-mortem

The blood and spleens collected post-mortem were cultured to detect *Y. pestis* on CIN agar and BHI with API20E identification (Rasoamanana et al., 1996). To identify colonies that morphologically did not resemble *Y. pestis*, MALDI-TOF (Bruker Biotyper) identification was also conducted, according to the manufacturer’s instructions. In brief, bacterial colonies were collected and extracted with an organic solvent comprising 50% acetonitrile, 47.5% water, and 5% trifluoroacetic acid with vigorous mixing for 20 min, followed by 5 min of incubation at room temperature. Subsequently, the extracted samples were centrifuged, and 1 μL aliquots were analyzed by MALDI-TOF to confirm the bacterial species.

### Statistical analysis

Graphs were generated using the software GraphPad PRISM (V9.0). Antibody titer data from initial experiments was log-transformed and analyzed by a linear model. The validity of the analysis against assumptions was assessed using standard diagnostic plots. Pairwise comparisons were made using Bonferroni’s correction for familywise error.

Competition ELISA data were analyzed by a linear model where dilution and construct were included as non-continuous variables. The validity of the analysis against assumptions was assessed using standard diagnostic plots. Pairwise comparisons were made using Bonferroni’s correction for familywise error.

Antibody titer data from immunogenicity studies on the mixed saRNA was log-transformed and analyzed using multiple *t*-tests. The data were split by sample time and by antigen. The validity of the analysis against assumptions was assessed using Levene’s tests for unequal variance and a QQ plot. Bonferroni’s correction factor of 4 was used to account for familywise error. Survival was compared using IBM SPSS and log-rank tests.

## Results

### Preparation and down-selection of DNA constructs

**Figure 1:**
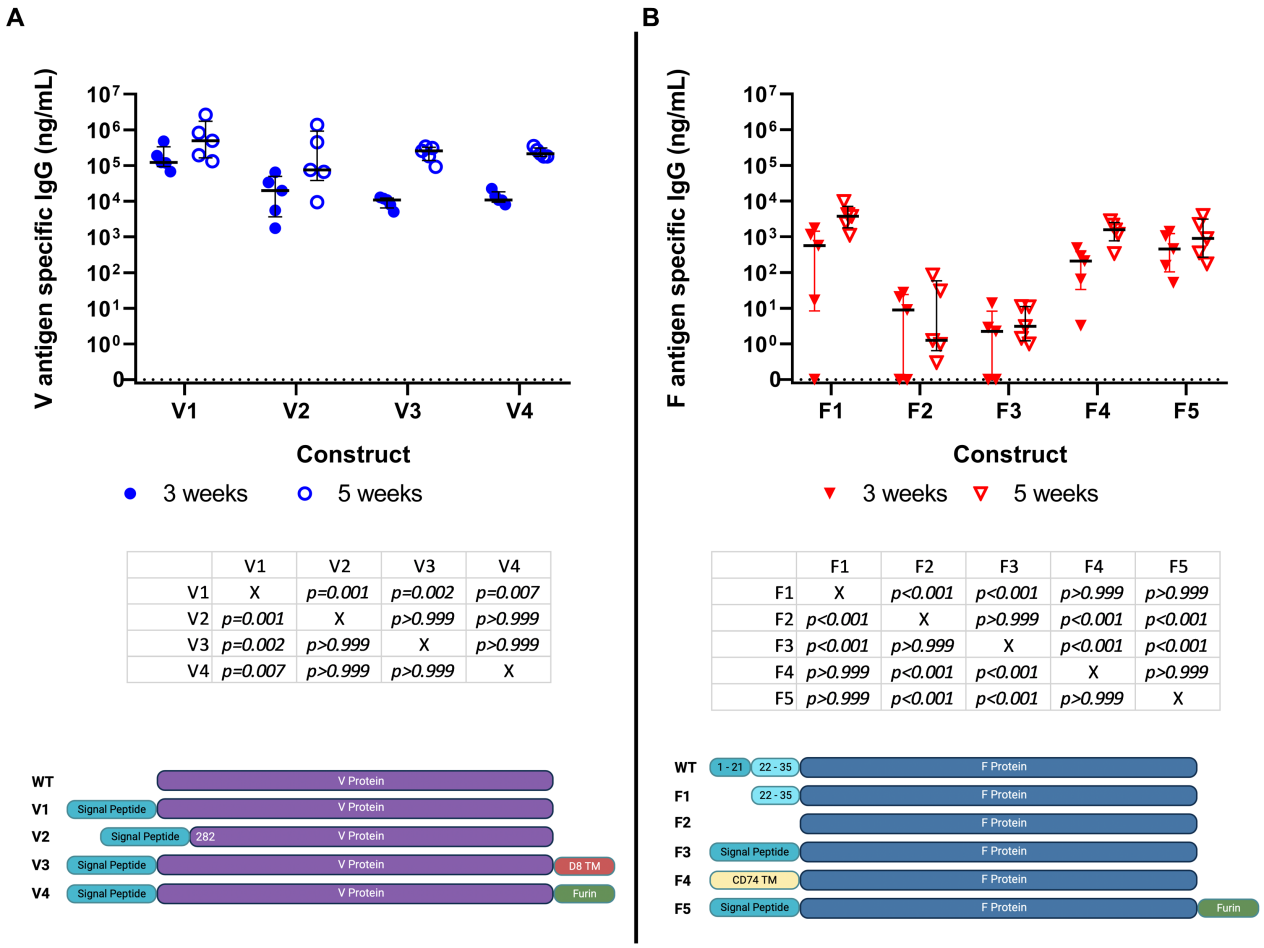
Specific IgG titer induced by immunization of Balb/c mice (5 per group) with 9 different DNA constructs encoding saRNA encoding V (4 constructs) (panel **A**) and F1 (5 constructs) (panel **B**) antigens; serum IgG titers at weeks 3 and 5 post-immunization are shown in ng/ml. No cross-reactivity to the antigens was detected in day 0 blood samples. These data were analyzed using linear models. Individual comparisons between constructs were made using Bonferroni’s post-tests, including both time points. These tests are summarized below each figure. The graphic under the figure compares the designs of the constructs.

Previous studies have shown that DNA constructs for F1 and V were immunogenic in mice (Leary et al., 1995; Grosfeld et al., 2003). For this reason, an initial screen was performed using DNA encoding variations of the sequences for F1 (*n* = 5) and V (*n* = 4), each administered IM with electroporation to groups of five female BALB/c mice. All the DNA constructs were shown to be immunogenic at 3 weeks, with an increased titer at week 5, 1 week after boosting (Figure 1).

At 3 weeks, mice had developed median anti-F1 IgG titers of 0.5 μg/mL, rising to 5 μg/mL by 5 weeks. The corresponding values for anti-V titers were 100 μg/mL, rising to 500 μg/mL. These data were analyzed using statistical models. With regard to the responses to the four V constructs, we found evidence that titers increased over time (*p* < 0.001) and that there were different amplitudes of response against the different constructs (*p* < 0.001). There was no evidence for the different constructs having an altered response at different times (*p* = 0.146). Post-tests indicated that the V1 construct elicited a stronger response when compared to the others (*p* < 0.01). With regards to the responses to the five F1 constructs, we found evidence that titers increased over time (*p* = 0.004) and that there were different amplitudes of response against the different constructs (*p* < 0.001). There was no evidence for the different constructs having an altered response at different times (*p* = 0.441). Post-tests indicated that the F1, F4, and F5 constructs elicited higher responses than the F2 and F3 constructs. Of these, the F1 construct was the most authentic representative of wild-type protein. Of the four V constructs, V1 appeared to be the most immunogenic.

Thus, V1 and F1 (construct 1) were down-selected for the derivation of saRNA constructs and testing in a further immunogenicity study.

**Figure 2:**
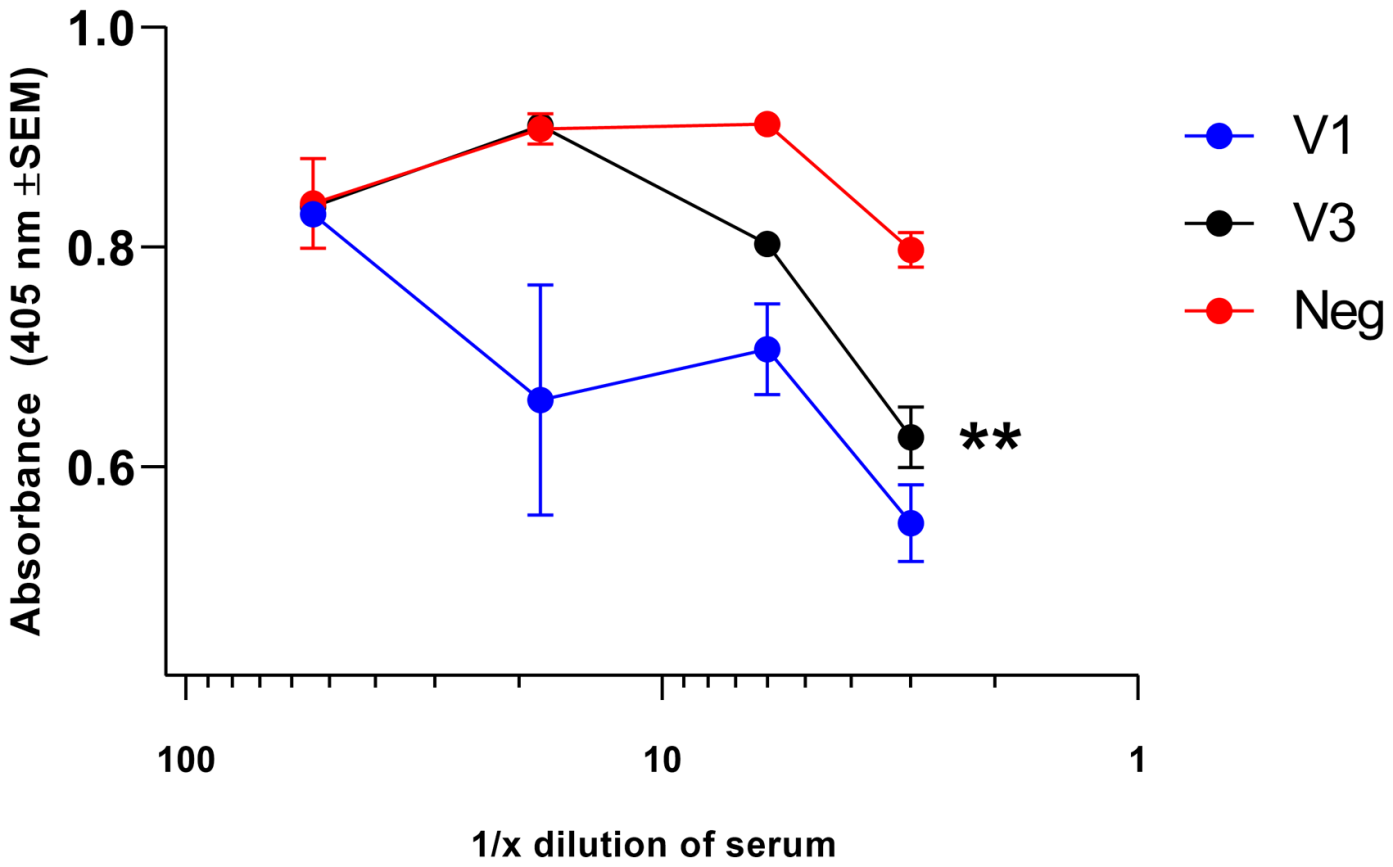
Assay of NAb directed toward the V antigen by competitive ELISA. Test sera, collected at week 5 of the immunization schedule, were pooled and then diluted in PBS in the range of 1:5 to 1:2000 before use. At low dilution (<1:100), they competed with 80 ng of the neutralizing biotinylated Mab 7.3 for binding to the V antigen, as detected with anti-mouse IgG streptavidin peroxidase, indicating the induction of NAb. Neutralizing activity was greater in sera from the V1-immunized mice compared with the V3-immunized group. The significance marker indicates a difference in titer compared to the negative control using the interaction term of a linear model (** shows *p* < 0.01).

The appropriateness of down-selection of the V1 construct was further confirmed by assaying for NAb. Within the total antibody response to V, the NAb component was assayed in test sera from immunized BALB/c mice, pooled by the treatment group (Figure 2). Test sera from mice immunized with construct V1 competed with Mab 7.3 for binding when the test sera were concentrated (<1:100), as indicated by the reduction in OD signal. By comparison, sera from mice immunized with construct V3 competed with Mab 7.3 only when diluted <1:10. The data indicated that construct V1 was significantly more potent than construct V3, and therefore, V1 was taken forward for the efficacy study, together with the most immunogenic F1 construct (hereafter denoted F1).

These data were analyzed using statistical models. Evidence was found for a dilution effect (*p* < 0.001), a difference between vaccinations (*p* < 0.001), and an interaction between the two (*p* = 0.007). This interaction was critical due to the different background optical densities for the three conditions, and the interaction represents the altered response to higher titers. Post-tests using the interaction term indicated no evidence for a V3 effect (*p* = 0.110), but there was evidence of competition by V1 (*p* < 0.002).

### Immunogenicity screening of saRNA constructs

**Figure 3:**
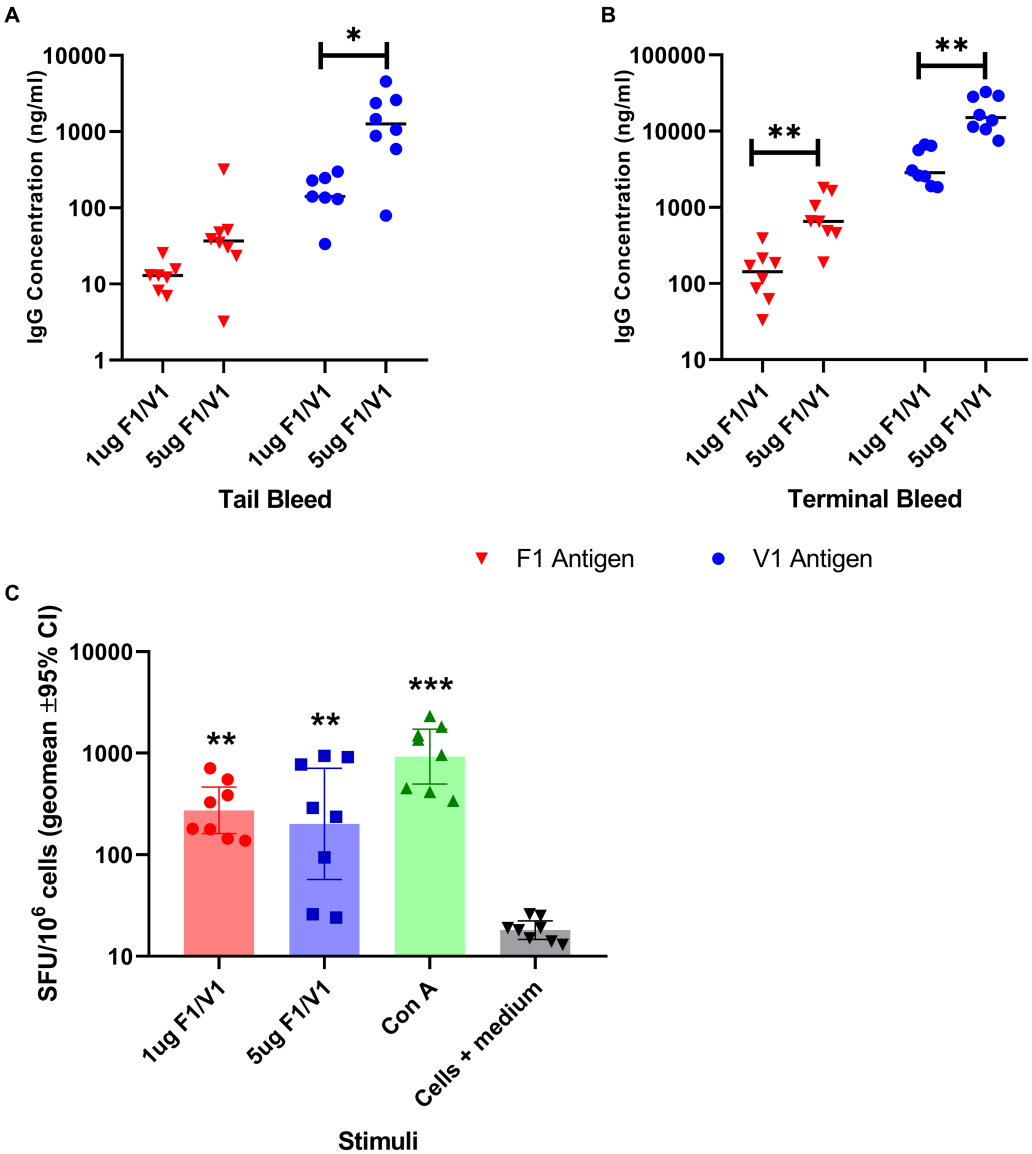
Specific IgG titer induced by immunization of BALB/c mice (8 per group) with saRNA encoding V and F1 antigens; serum IgG titers at week 4 (tail bleed, panel **A**) and 6 post-immunization (terminal, panel **B**) are shown in ng/ml. The bar indicates the sample median, and each data point represents a single mouse. Panel **C**: IFN-gamma-producing splenocytes from Balb/c mice (*n* = 8) immunized by saRNA encoding V and F1 antigens; cells were restimulated by two different doses of a mixture of V and F1 antigens or concanavalin A mitogen. The bar indicates the sample median in terms of spot-forming units (SPU) per 10^6^ splenocytes, and each data point represents a single mouse. The significance marker indicates a difference in titer compared to the negative control using Bonferroni’s corrected *T*-tests (* shows *p* < 0.05 and ** shows *p* < 0.01). For (panel **C**), significance markers indicate the results of a Dunn’s post-test comparing each stimulated group to unstimulated cells (** is *p* < 0.01 and *** is *p* < 0.001).

The individual saRNA constructs, encoding F1 or V1 and formulated in LNP, were combined to immunize groups of eight female BALB/c mice. The mice were immunized at two different dose levels (1 μg and 5 μg) by the i.m. route and developed dose-related antibody titers to each antigen, which increased 10-fold over the 35 day study period (Figures 3A,B). These data were analyzed using t-tests. Evidence was found for vaccine dose-enhancing response in F1 terminal bleeds (*p* < 0.004), V terminal bleeds (*p* < 0.004), and V tail bleeds (*p* = 0.012), but not F1 tail bleeds (*p* = 0.171).

Splenocytes collected at the end of the study were tested *in vitro* for a recall response to F1 and V, which showed that immunized mice also developed antigen-specific T cell responses to the combined F1 and V antigens (Figure 3C), which were significantly greater (*p* < 0.01) than the cells+medium only response.

### Immune response in OF1 mice

Prior to the pathogen challenge, groups of OF1 (outbred) mice were immunized with the F1 and V1 constructs in combination and then monitored for titer development at days 0, 23, and 51. The mice were challenged with *Y. pestis* s.c. at day 56. The number of mice per group positive for titer to F1 at each time point was recorded in Table 2 so that most vaccinated mice had detectable antibodies to F1 by day 51. The effect of the challenge was to boost anti-F1 titers and induce an anti-F1 response in some animals in the negative control groups. Surprisingly, in two animals (one each from groups 2 and 6), we noted that the effect of the challenge was to render these seronegative for F1. We had not previously sampled mice post-challenge for antibody titer and so had not previously observed this.

**Table 2:** Seropositivity in OF1 mice pre-and post-challenge.

Vaccine	Anti-F1 (day 0)	Anti-F1 (day 23)	Anti-F1 (day 51)	No dead pre-challenge	No. challenged at day 56	Positive anti-F1 (day 60)	Anti-V (day 0)	Positive anti-V (day 60)	Survivors at d. 70
RNA 1 μg F1 + RNA 1 μg V in LNP	Neg*	5/7	6/7	1	7	5/7 (2 dead before sample)	Neg^	5/7	5/7
RNA 5 μg F1 + RNA 5 μg V in LNP	neg	4/7	6/7	1	7	5/7 (1 dead before sample; 1 became seronegative)	neg	6/7	6/7
RNA 10 μg Ha in LNP	neg	0/8	0/8	0	8	1/ 3 (5 dead before sample)	neg	neg	1/8
RNA 1 μg F1 + RNA 1 μg V in LNP	neg	0/7	2/7	1	7	5/7 (2 dead before sample)	neg	Not done	5/7
RNA 5 μg F1 + RNA 5 μg V in LNP	neg	0/8	5/8	0	8	5/5 + 3 dead before sample)	neg	5/5	5/8
5 μg F1+ 5 μg V/20% alhydrogel	neg	8/8	8/8	0	8	7/8 (1 became sero negative)	neg	7/8	7/8
RNA 10 μg Ha in LNP	neg	0/7	0/7	1	7	1/7 (+6 dead before sample)	neg	neg	1/7
Alhydrogel	neg	0/8	0/7	1	7	1/4 (3 dead before sample)	neg	neg	1/7

Mice were sampled at days 0, 23, and 51 pre-challenge and at day 60 (4 days post-challenge), and antibody titers to F1 and V were determined by standard ELISA where a positive titer to F1 is >0.15 OD_414_ and a positive titer to V is >0.78 OD_414_. The number of mice per group surviving the challenge at day 70 is recorded. * Negative titer for F1 is <0.15 OD_414_ and ^ negative titer for V is <0.78 OD_414_.

The number of challenged mice positive for antibodies to V at day 60 correlated closely to the number of survivors per group at day 70. In vaccinated groups, increases in anti-V titers at day 60 post-challenge, compared with the day 0 baseline OD_414_ provided by the pre-immunization sera, were sevenfold to ninefold (Table 3).

**Table 3:** Fold increase in titers to V in OF1 mice at day 60.

Vaccine	Treatment	No. tested at day 60	Mean anti-V titer in OD units at day 60 post-challenge ± sem	Fold increase in titers at day 60 over baseline (0.39)
1. RNA vaccine, 1 μg	RNA 1 μg F1 + RNA 1 μg V in LNP	3	3.38 ± 0.13	8.7
2. RNA vaccine, 5 μg	RNA 5 μg F1 + RNA 5 μg V in LNP	6	3.27 ± 0.17	8.39
3. Negative control	RNA 10 μg Ha in LNP	N.D.		
4. RNA vaccine, 1 μg	RNA 1 μg F1 + RNA 1 μg V in LNP	3	3.13 ± 0.09	8.03
5. RNA vaccine, 5 μg	RNA 5 μg F1 + RNA 5 μg V in LNP	5	3.34 ± 0.36	8.56
6. Recombinant F1 and V vaccine, 5 μg	5 μg F1+ 5 μg V/20% alhydrogel	6	3.05 ± 0.05	7.82
7. Negative control	RNA 10 μg Ha in LNP	1	1.76 (*n* = 1)	4.51
8. Negative control	Alhydrogel	1	0.47 (*n* = 1)	1.2

Mice were sampled at day 60 (4 days post-challenge), and antibody titers to V were determined by standard ELISA and recorded as OD_414_ units. The fold increase in titers pre-immunization to post-challenge was determined. N.D. is not done. Pre-immunization sera had a baseline OD_414_ of 0.39. A positive titer to V is >0.78 OD_414._ Therefore, a positive fold increase in titer is >2.0.

### Pre-challenge observations

After immunization and before boosting and subsequent challenge, there were some spontaneous single deaths in some groups, i.e., on day 3 (group 1), on day 7 (group 2), on day 8 (negative control group 7), on day 17 (group 4), and on day 38 (alhydrogel negative control group 8) (Table 2). These deaths occurred in the RNA-negative and alhydrogel control groups as well as in the immunized groups and were not chronologically related to the procedure or a vaccine. At post-mortem, spleen homogenates from the affected animals were cultured on CIN agar and in BHI medium, and none showed typical *Y. pestis* colonies (Table 4). The individual mice that died pre-challenge in groups except group 7 showed Gram-negative colony growth on CIN agar. *E. coli* was confirmed by MALDI-TOF in the individual mouse from group 8.

**Table 4:** Comparison of times to death p.i. with *Y. pestis* for vaccinated and negative control groups, showing the statistically significant impact of vaccination on survival.

Treatment	Treatment group and mouse	No.per group dead p.i. with *Y. pestis*	Day of death p.i. with *Y. pestis* (where survivors were culled on day 14 p.i.)	Mean time to death ± sem	Significance of difference compared to relevant control
1 μg RNA vaccine; 2.8 MLD *Y. pestis*	1.1	2/7	14	11.00 ± 1.80	*p* = 0.039
	1.2		3		
	1.3		14		
	1.4		14		
	1.5		6		
	1.6		14		
	1.7		14		
5 μg RNA vaccine; 2.8 MLD *Y. pestis*	2.1	1/7	14	12.43 ± 1.46	*p* = 0.010
	2.2		14		
	2.3		14		
	2.4		14		
	2.5		14		
	2.6		3		
	2.7		14		
10 μg irrelevant RNA; 2.8 MLD *Y. pestis*	3.1	7/8	6	5.38 ± 1.22	n/a
	3.2		3		
	3.3		14		
	3.4		4		
	3.5		3		
	3.6		6		
	3.7		4		
	3.8		3		
1 μg RNA vaccine; 28 MLD *Y. pestis*	4.1	2/7	14	11.00 ± 1.80	*p* = 0.022
	4.2		14		
	4.3		3		
	4.4		14		
	4.5		14		
	4.6		4		
	4.7		14		
5 μg RNA vaccine; 28 MLD *Y. pestis*	5.1	3/8	14	9.88 ± 1.88	*p* = 0.066
	5.2		14		
	5.3		3		
	5.4		3		
	5.5		14		
	5.6		3		
	5.7		14		
	5.8		14		
5 μg F1 + 5 μg V Alhydrogel; 28 MLD *Y. pestis*	6.1	1/8	14	12.88 ± 1.05	*p* = 0.002
	6.2		14		
	6.3		14		
	6.4		14		
	6.5		14		
	6.6		5		
	6.7		14		
	6.8		14		
10 μg irrelevant RNA; 28 MLD *Y. pestis*	7.1	6/7	3	4.57 ± 1.46	n/a
	7.2		3		
	7.3		3		
	7.4		3		
	7.5		3		
	7.6		14		
	7.7		3		
Alhydrogel; 28 MLD *Y. pestis*	8.1	6/7	3	4.86 ± 1.42	n/a
	8.2		3		
	8.3		4		
	8.4		3		
	8.5		4		
	8.6		14		
	8.7		3		

### Enumeration of the challenge dose levels

Retrospective culture determined the challenge dose levels delivered in a total volume of 0.1 mL/mouse to groups 1–3 as 180 cfu/mouse s.c. and to groups 4–8 as 1800 cfu/mouse s.c., representing 2.8 and 28 MLD, respectively.

### Survival

**Figure 4:**
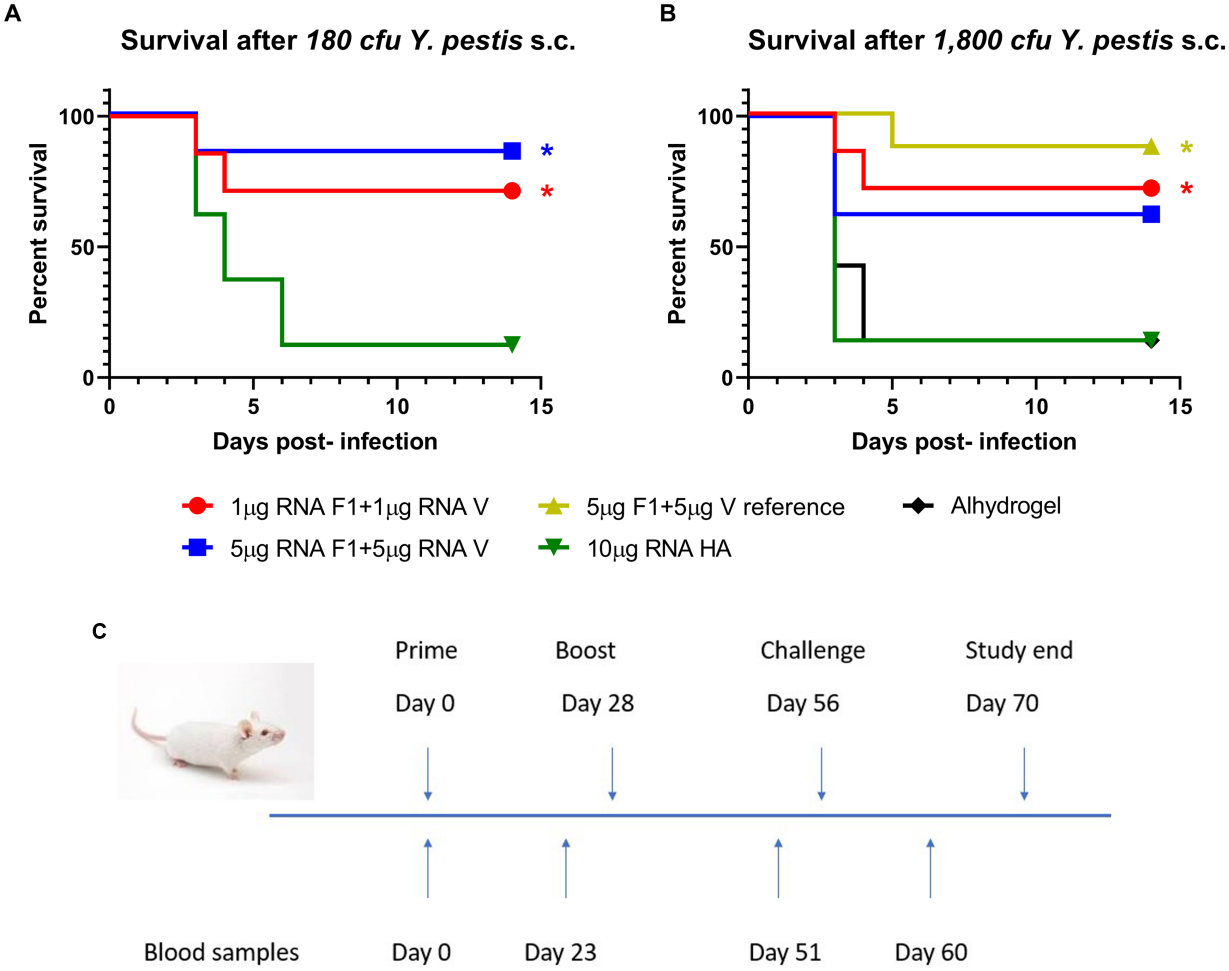
Survival. Groups of mice immunized on days 0 and 28 were challenged s.c. with *Y. pestis* 10-21/S on day 56 and observed for 14 days p.i. Survival in mice challenged with 180 cfu (panel **A**) or 1800 cfu (panel **B**) is shown. Significance markers are indicative of log-rank tests comparing each vaccinated group to its relevant control. * implies *p* < 0.05. (Panel **C**) shows the study timeline.

All surviving immunized mice were challenged s.c. with *Y. pestis* 10-21/S on day 56 of the schedule and monitored for 14 days p.i. Two target dose levels of *Y. pestis* were used to challenge the mice: 10^2^ or 10^3^ cfu, and the survival curves are shown below (Figures 4A,B). Panel Figure 4C summarises the immunisation schedule prior to challenge.

At the lower dose level (180 cfu *Y. pestis*), 1 μg or 5 μg of RNA vaccine protected 5/7 mice, compared with 1/8 survivors in the negative control group. At the higher challenge level (1,800 cfu), 5/7 mice or 5/8 mice immunized with 1 μg or 5 μg of the RNA vaccine respectively, survived the challenge compared with 7/8 mice in the positive control; in the negative control groups, there was 1 surviving mouse in each.

Analysis of time to death (TTD) p.i. with *Y. pestis* showed a significant difference between the vaccinated groups and their matched negative controls (Table 4). Negative control mice (7/8) that were given 2.8 MLD were dead at 5.38 ± 1.22 days, whereas 12/14 negative control mice receiving 28 MLD were dead before day 5. In contrast, 21/29 (73%) of RNA-vaccinated mice survived for 14 days. At the lower challenge level, the significant difference between TTD for OF1 mice vaccinated with 1 μg or 5 μg saRNA and their matched negative controls was *p* = 0.039 and *p* = 0.01, respectively. At the higher challenge level, there was a significant difference in TTD for saRNA-vaccinated mice at the 1 μg dose level compared with their matched negative controls (*p* = 0.022).

### Bacteriology post-mortem

The results of bacteriological analysis at post-mortem in mice challenged with *Y. pestis* clearly show the effect of saRNA vaccination on the clearance of *Y. pestis* from the spleens, with 5/7 or 6/7 vaccinated mice in groups 1 and 2 culture-negative on CIN agar for *Y. pestis* after the challenge with 2.8 MLD (Table 5). By comparison, only 1/8 mice in the negative control group 3 were culture-negative for *Y. pestis*. In the groups challenged with 28MLD *Y. pestis*, 5/7 or 5/8 vaccinated mice in groups 4 and 5 were culture-negative for *Y. pestis* compared with 1/7 in each of the matched negative control groups. By comparison, 7/8 mice in the reference vaccine group were culture-negative for *Y. pestis* after challenge with 28MLD.

**Table 5:** Post-mortem bacteriological analysis on CIN agar showing *Y. pestis* colonies and MALDI-TOF analysis of atypical colonies, where CNC = colonies on CIN agar that are not *Y. pestis*; and FCNR = fine, small red colonies typical of *Y. pestis*.

Treatment	Deaths pre-challenge: PM spleen culture on CIN agar	No. per group with *Y. pestis* colonies on CIN agar p.i.	The density of *Y. pestis* growth on CIN agar in (*n*) mice with day of death p.i.	No. per group without *Y. pestis* on CIN agar p.i.	No. per group with growth on BHI medium (*Y.pestis*) with day of death p.i.	MALDI-TOF on atypical colonies on CIN agar with day of death p.i.
1 μg RNA vaccine; 2.8 MLD *Y. pestis*	1/8; CNC ++	2/7	FCNR+++ (2) +d.3, +d.6	5/7	2/7 +d3,+d6	*K.pnuemoniae* 1/5, +d.6
5 μg RNA vaccine; 2.8 MLD *Y. pestis*	1/8: CNC++	1/7	FCNR+++ (1) +d.3	6/7	1/7 +d.14	
10 μg irrelevant RNA; 2.8 MLD *Y. pestis*	None	7/8	FCNR+++ (7) + d.3–6	1/8	3/8 +d.4,+d6,+d6	*E.coli* 1/1,+d.6
1 μg RNA vaccine; 28 MLD *Y. pestis*	1/8: CNC+	2/7	FCNR+++ (2) +d.3–4	5/7	0/7	
5 μg RNA vaccine; 28 MLD *Y. pestis*	None	3/8	FCNR+++ (3) + d.3	5/8	0/8	
5 μg F1 + 5 μg VAlhydrogel; 28 MLD *Y. pestis*	None	1/8	FCNR+++ (1) +d.5	7/8	2/8 +d5, +d14*	
10 μg irrelevant RNA; 28 MLD *Y. pestis*	1/8: no growth	6/7	FCNR+++ (6) +d.3	1/7	2/7 +d3, +d14*	*E.coli* 1/1, +d.3
Alhydrogel; 28 MLD *Y. pestis*	1/8: CNC++ *E.coli* confirmed	6/7	FCNR+++ (6) +d.3–4	1/7	0/7	

Growth in the BHI medium was also identified as *Y. pestis,* with two exceptions (denoted *).

Where *Y. pestis* growth was found on CIN agar, this was also confirmed by culture of spleen homogenates in BHI medium, with two exceptions. Individual surviving mice (one each from group 6 (sub-unit vaccine) and group 7 (negative control)) had positive growth on BHI, which was not *Y. pestis*, determined when culled at day 14 p.i. (Table 5).

These data show a significant influence of vaccination on the clearance of *Y. pestis* from the spleens. MALDI-TOF analysis on bacterial colonies other than *Y. pestis* growing on CIN agar showed *Klebsiella pneumoniae* in one mouse from the RNA vaccine group 1 and *E.coli* in one mouse from the negative control group 3 and in one mouse from the negative control group 7.

## Discussion

BALB/c mice immunized with a DNA construct for V responded with the secretion of functional antibodies. The latter competed with the neutralizing Mab7.3 for binding to V antigen *in vitro*. This is significant since Mab7.3 recognizes a key conformational epitope on the V antigen and has also been shown to protect mice, both on administration before and after their exposure to *Y. pestis* (Andrianaivoarimanana et al., 2021). These results strongly suggest that the delivery of a nucleotide vaccine resulted in a conformationally authentic production of the V antigen *in vivo*, with the neutralizing epitope preserved. BALB/c mice subsequently immunized with the down-selected saRNA constructs for F1 and V demonstrated 100% seroconversion, and also developed a significant *in vitro* recall response to the combined antigens, as determined by the ELISpot assay. The CMI response did not increase with vaccine dose level, a facet of CMI that was previously noted (Billeskov et al., 2019).

The OF1-outbred mice also responded to immunization with the down-selected saRNA constructs with the induction of specific IgG for F1 and V. While vaccine dose level resulted in a significant difference in antibody titer to both F1 and V in BALB/c mice, this was not the case for the OF1 mice, and the antibody titer was slower to develop in the OF1 mice. However, at day 60, 4 days p.i., the OF1 mice had antibody titers to V that were not significantly different from those given the reference vaccine at the 5 μg dose level. Regarding the challenge with a contemporary Malagasy clinical isolate of *Y. pestis*, 70–80% protection was achieved with as little as 1 μg saRNA for each antigen. Complete protection was achieved in those individual animals with greater antibody responses. In addition, at the post-mortem of challenged mice, we observed a significant effect of vaccination on the clearance of *Y. pestis* from the spleens, with the majority of mice in the vaccinated groups being culture-negative, whereas the majority of mice in the negative control groups were culture-positive for *Y. pestis*. Again, however, in the OF1 mice, there was no significant effect of vaccine dose level on bacteriological clearance, although vaccinated groups had much greater clearance overall than the negative control groups. This illustrates the point that the OF1 mice are much more heterogeneous in their responses than the BALB/c mice.

In these initial immunogenicity and efficacy studies, we were seeking proof of principle for this novel vaccine. Thus, the challenge levels used were relatively modest, with a 10-fold increment from 180 cfu to 1800 cfu, which resulted in one surviving mouse in each negative control group at either challenge level. Now that we have demonstrated the efficacy of the saRNA vaccine in terms of both survival and clearance of *Y. pestis* p.i., we will progress to more discriminatory challenges with enhanced vaccine constructs and further examination of vaccine dose levels in the future. The above data indicate that 1–5 μg of these saRNA constructs provide a baseline from which to compare the efficacy of future constructs.

Nevertheless, the efficacy data generated in outbred mice with more heterogeneous responses are encouraging. The increase in titers from baseline, determined in the OF1 mice at day 60 and 4 days p.i., was less than 10-fold. In this study, BALB/c mice were not challenged, thus an exact comparison is not possible. However, through the immunization schedule, BALB/c mice showed increases in anti-V titers of >10-fold. Indeed, the level of protection observed in the OF1 mice was impressive, given the lower titers recorded in the OF1 mice relative to the BALB/c mice. These data validate the immunogens presented by the saRNA vector, and we would expect even more robust protection if antibody responses were more analogous to BALB/c. However, an analysis of TTD data for vaccinated and negative control OF1 groups demonstrated a statistically significant effect of vaccination on survival. These preliminary data indicate efficacy for the saRNA vaccine in the bubonic plague model, although at this stage, we do not fully understand the difference in response between the OF1 and BALB/c models.

Nevertheless, screening vaccines in outbred and inbred mice is highly desirable, as outbred mice may provide a more realistic indication of the protective efficacy achievable as vaccines are translated to other species and eventually to the clinic (Mogil et al., 2018).

In the 56 day vaccination period, 5/64 OF1 mice died, which could not be attributed to any specific cause and may be a facet of the difficulties of maintaining a colony of outbred mice in the environmental conditions prevailing in Madagascar. These mice were not bred under specific pathogen-free conditions, and therefore, it is not unexpected that they should have common Gram-negative contaminants in their spleens, such as *E.coli*, as identified by bacteriological analysis post-mortem. However, when the OF1 mice were challenged with *Y. pestis*, the hazard rate for groups that received no immunization rapidly increased, and 19 of 22 mice succumbed to disease over the 14 day p.i. period. A comparison of mortality pre-and post-challenge indicates a 75-fold increase in odds (15.12–272.2 95% CIs). This increase in mortality was clearly driven by the addition of highly pathogenic bacteria.

Recently, a mRNA vaccine based on a circularly permutated F1 has been reported to provide 100% protective immunity in mice against the fully virulent *Y. pestis* KIM53 (100LD_50_) in the bubonic model (Kon et al., 2023). This construct encoded a monomeric F1 conjugated to a human Fc signal peptide and was protective after only a single dose. However, the authors recognize that this approach would not protect against acapsular strains of *Y. pestis*, which retain virulence. In this study, we encoded both F1 and V, the essential virulence proteins for plague, in our saRNA vaccine, and we have demonstrated significant efficacy against a contemporaneous, virulent clinical isolate of *Y. pestis* from Madagascar. Since we have demonstrated that this saRNA vaccine induced specific antibody and recall responses to each of F1 and V, we predict that it would provide efficacy against acapsular strains of plague as well as encapsulated strains, an aspect that will be tested in future studies.

The major benefit of RNA vaccines over protein sub-unit vaccines is the ease of manufacture and the more streamlined downstream processing compared with protein sub-unit vaccines. For the individual protein sub-units, F1 is relatively easy to isolate and purify from an *E.coli* fermentation, but V is rather less so, as, in nature, it is an intracellular protein in *Y. pestis* until contact is made with the host cell when it is translocated through the injectisome. Downstream processing of the sub-units is labor-intensive and expensive, so the RNA vaccine, particularly in the self-amplifying form used here, provides dual benefits in terms of simplified manufacture and immunogenicity. Here, we have shown the RNA vaccine to provide significant protection compared with matched negative controls, although the current prototype is not quite as efficacious as the F1 + V sub-unit vaccine used here as a reference vaccine.

Studies are planned to further optimize the existing constructs to increase potency and efficacy. While we have chosen here to use individual saRNA constructs for F1 and V, a single launch saRNA construct from which both F1 and V can be expressed is also feasible and will be evaluated. The data generated to date are significant in that, to our knowledge, it is the first time that saRNA constructs for a serious bacterial pathogen have been shown to be immunogenic and efficacious against a fully virulent strain of *Y. pestis* in the murine bubonic model. While promising, direct translation to humans cannot be assured. Although the first clinical trials of saRNA vaccines against COVID-19 have shown this platform to have good safety and tolerability profiles, induced antibody responses were lower than predicted in small animal studies (McKay et al., 2020; Szubert et al., 2023). Further optimisation of saRNA for human use is ongoing, including improved manufacturing processes, strategies to subvert innate responses that may restrict expression, and optimized formulation strategies (Blakney et al., 2021a,b). In the next phase of research, we will test existing and enhanced constructs in a pneumonic challenge model with the objective of achieving a novel yet efficacious vaccine for plague.

The concept and realization of an saRNA vaccine for plague is an exciting prospect since this would be relatively fast to manufacture and thus develop for clinical approval, at least to emergency use status, in which context this low-dose platform could be rapidly fielded to deal with a disease outbreak. Finally, the elicitation of significant specific cell-mediated immunity in mice by the saRNA constructs used here is a positive aspect of this vaccine approach, indicating, as it does, the induction of immune memory.

## Data availability statement

The datasets presented in this study can be found in online repositories. The names of the repository/repositories and accession number(s) can be found in the article/Supplementary material.

## Ethics statement

The animal studies were approved by Institut Pasteur de Madagascar IACUC. The studies were conducted in accordance with the local legislation and institutional requirements. Written informed consent was obtained from the owners for the participation of their animals in this study.

## Author contributions

RS, PM, and EW conceptualized the vector and study design. PM, KS, and PR performed the *in vitro* experiments. PM, VM, and KS, performed the *in vivo* studies at ICL. VA, MR, and LR performed the *in vivo* challenge experiments at IPM. EW, TL, and RS analyzed the data and wrote the manuscript with constructive feed-back from all authors. All authors contributed to the article and approved the submitted version.
