# Supplementary material for: A self-amplifying RNA vaccine provides protection in a murine model of bubonic plague
Source: Front Microbiol. 2023 Nov 2;14:1247041. doi: 10.3389/fmicb.2023.1247041 (PMC10652872; doi:10.3389/fmicb.2023.1247041)
Supplement: Supplementary file 1 [file Data_Sheet_1.PDF]

Construct Sequences (**Bold** – Start and Stop Codons)

F1

ATGGCTGATCTGACAGCCAGCACAACAGCCACCGCCACACTGGTGGAACCTGCCAGAATCACCCTG
ACCTACAAAGAGGGCGCTCCCATCACCATCATGGACAACGGCAACATCGACACCGAGCTGCTCGTG
GGCACACTGACACTCGGCGGATACAAGACCGGCACCACAAGCACCAGCGTGAACTTCACAGATGCC
GCTGGCGACCCCATGTACCTGACCTTCACAAGCCAGGACGGCAACAACCACCAGTTCACCACCAAAG
TGATCGGCAAGGACAGCCGGGACTTCGACATCAGCCCCAAAGTGAATGGCGAGAACCTCGTGGGC
GACGATGTGGTGCTTGCTACAGGCAGCCAGGACTTCTTCGTGCGGAGCATCGGAAGCAAAGGCGG
AAAACTGGCCGCTGGCAAGTACACCGATGCCGTGACAGTGACCGTGTCCAACCAG**TGA**

F2

ATGGAACCTGCCAGAATCACCCTGACCTACAAAGAGGGCGCTCCCATCACCATCATGGACAACGGC
AACATCGACACCGAGCTGCTCGTGGGCACACTGACACTCGGCGGATACAAGACCGGCACCACAAGC
ACCAGCGTGAACTTCACAGATGCCGCTGGCGACCCCATGTACCTGACCTTCACAAGCCAGGACGGCA
ACAACCACCAGTTCACCACCAAAGTGATCGGCAAGGACAGCCGGGACTTCGACATCAGCCCCAAAG
TGAATGGCGAGAACCTCGTGGGCGACGATGTGGTGCTTGCTACAGGCAGCCAGGACTTCTTCGTGC
GGAGCATCGGAAGCAAAGGCGGAAAACTGGCCGCTGGCAAGTACACCGATGCCGTGACAGTGACC
GTGTCCAACCAG**TGA**

F3

ATGGATAGAGCCAAACTGCTGCTGCTCCTGTTGCTCCTCCTGCTGCCTCAGGCTCAGGCTGTGGAAC
CTGCCAGAATCACCCTGACCTACAAAGAGGGCGCTCCCATCACCATCATGGACAACGGCAACATCGA
CACCGAGCTGCTCGTGGGCACACTGACACTCGGCGGATACAAGACCGGCACCACAAGCACCAGCGT
GAACTTCACAGATGCCGCTGGCGACCCCATGTACCTGACCTTCACAAGCCAGGACGGCAACAACCAC
CAGTTCACCACCAAAGTGATCGGCAAGGACAGCCGGGACTTCGACATCAGCCCCAAAGTGAATGGC
GAGAACCTCGTGGGCGACGATGTGGTGCTTGCTACAGGCAGCCAGGACTTCTTCGTGCGGAGCATC
GGAAGCAAAGGCGGAAAACTGGCCGCTGGCAAGTACACCGATGCCGTGACAGTGACCGTGTCCAA
CCAG**TGA**

F4

ATGAAGTGTTCTAGAGGCGCCCTGTACACCGGCTTCAGCATCCTGGTTACACTGCTGCTGGCCGGAC
AGGCTACCACCGCCTATTTTCTGTATGGCTCTGGCGCCGATCTGACCGCCTCTACAACAGCCACAGCC
ACACTGGTGGAACCCGCCAGAATCACCCTGACCTACAAAGAGGGCGCTCCCATCACCATCATGGACA
ACGGCAACATCGACACCGAGCTGCTCGTGGGCACACTGACACTCGGCGGATACAAGACCGGCACCA
CAAGCACCAGCGTGAACTTCACAGATGCCGCTGGCGACCCCATGTACCTGACCTTCACAAGCCAGGA
CGGCAACAACCACCAGTTCACCACCAAAGTGATCGGCAAGGACAGCCGGGACTTCGACATCAGCCC
CAAAGTGAATGGCGAGAACCTCGTGGGCGACGATGTGGTGCTTGCTACAGGCAGCCAGGACTTCTT
CGTGCGGAGCATCGGAAGCAAAGGCGGAAAACTGGCCGCTGGCAAGTACACCGATGCCGTGACAG
TGACCGTGTCCAACCAG**TGA**

F5

ATGGATAGAGCCAAACTGCTGCTGCTCCTGTTGCTCCTCCTGCTGCCTCAGGCTCAGGCTGTGGAAC
CTGCCAGAATCACCCTGACCTACAAAGAGGGCGCTCCCATCACCATCATGGACAACGGCAACATCGA
CACCGAGCTGCTCGTGGGCACACTGACACTCGGCGGATACAAGACCGGCACCACAAGCACCAGCGT
GAACTTCACAGATGCCGCTGGCGACCCCATGTACCTGACCTTCACAAGCCAGGACGGCAACAACCAC
CAGTTCACCACCAAAGTGATCGGCAAGGACAGCCGGGACTTCGACATCAGCCCCAAAGTGAATGGC
GAGAACCTCGTGGGCGACGATGTGGTGCTTGCTACAGGCAGCCAGGACTTCTTCGTGCGGAGCATC
GGAAGCAAAGGCGGAAAACTGGCCGCTGGCAAGTACACCGATGCCGTGACAGTGACCGTGTCCAA
TCAAGGCGGAGGCTCTGGTGGATCTGGCGGCGATATCATCAAGCTGCTGAACGAGCAAGTGAACAA
AGAGATGAACAGCGCCAACCTGTACATGAGCATGAGCAGCTGGGCCTACACACACAGCCTTGATGG
CGCCGGACTGTTCCTGTTTGATCACGCCGCCGAGGAATACGAGCACGCCAAGAAGCTGATCATCTTC
CTGAACGAGAACAACGTGCCCGTGCAGCTGACCAGCATTTCTGCCCCTGAGCACAAGTTCGAGGGC
CTGACACAGATCTTCCAGAAGGCCTACGAACACGAACAGCACATCAGCGAGAGCATCAACAACATC
GTGGACCACGCCATTAAGAGCAAGGATCACGCCACCTTCAACTTTCTGCAGTGGTACGTGGCCGAG
CAGCACGAGGAAGAGGTGCTGTTCAAGGACATCCTGGACAAGATCGAGCTGATCGGAAACGAGAA
CCACGGCCTGTACCTGGCCGACCAGTACGTGAAGGGAATCGCCAAGAGCCGGAAGTCC**TGA**

V1

ATGGACAGGGCCAAGCTGCTGCTCCTGCTGCTGCTGCTGCTGCCACAGGCCCAGGCCGTTATG
ATTAGGGCCTACGAACAGAATCCACAGCACTTCATTGAGGACCTGGAGAAAGTGAGAGTGGAGCA
GCTGACCGGCCACGGGAGCTCAGTGCTGGAGGAGCTGGTGCAGCTGGTGAAGGACAAGAACATCG
ATATCAGTATCAAGTACGACCCTCGCAAGGACAGTGAGGTGTTTGCCAACAGGGTGATTACCGATG
ACATCGAGCTGCTGAAAAAGATCCTGGCCTACTTTCTGCCAGAGGATGCCATTCTGAAAGGAGGCCA
TTACGATAACCAGTTGCAGAATGGGATCAAGCGAGTGAAAGAGTTCCTGGAGAGCAGTCCCAACAC
CCAATGGGAACTGAGGGCCTTCATGGCCGTGATGCACTTTAGCCTGACCGCCGACCGAATTGACGA
TGACATTCTGAAGGTGATTGTCGATAGCATGAACCACCACGGCGACGCCCGAAGCAAGCTGAGGGA
GGAGCTGGCCGAGCTGACCGCCGAACTGAAGATCTACTCCGTGATCCAGGCCGAAATCAATAAGCA
CCTGTCCTCTAGCGGCACCATCAATATTCACGATAAGAGCATCAACCTGATGGACAAGAACCTGTAT
GGATACACTGACGAGGAAATCTTTAAGGCCAGTGCCGAGTACAAGATCCTGGAGAAGATGCCACAG
ACTACCATTCAGGTGGACGGCTCCGAGAAGAAGATCGTGTCTATCAAGGACTTCCTGGGGTCTGAA
AACAAGAGGACTGGCGCCCTGGGAAACCTGAAAAATTCCTACAGCTACAACAAGGATAACAACGAG
CTGTCCCACTTCGCCACCACCTGCAGTGATAAATCCAGACCTCTGAACGACCTGGTTAGTCAGAAAA
CTACCCAGCTGAGCGATATCACCTCTCGCTTCAATTCTGCCATCGAGGCCCTGAATCGCTTTATCCAG
AAGTACGACAGCGTGATGCAGAGGCTGCTGGACGATACCTCCGGCAAG**TGA**

V2

ATGGATAGAGCCAAACTGCTGCTGCTCCTGTTGCTCCTCCTGCTGCCTCAGGCTCAGGCCGTGATGA
TCAGAGCCTACGAGCAGAACCCTCAGCACTTCATCGAGGACCTGGAAAAAGTGCGCGTGGAACAGC
TGACAGGCCACGGAAGTAGCGTGCTGGAAGAACTGGTGCAGCTGGTCAAGGACAAGAACATCGAC
ATCAGCATTAAGTACGACCCTCGGAAGGACAGCGAGGTGTTCGCCAACAGAGTGATCACCGACGAC
ATCGAGCTGCTGAAGAAGATCCTGGCCTACTTCCTGCCTGAGGACGCCATCCTGAAAGGCGGCCACT
ACGACAATCAGCTGCAGAACGGCATCAAGCGCGTGAAAGAGTTCCTGGAAAGCAGCCCCAACACAC
AGTGGGAGCTGAGAGCCTTCATGGCTGTGATGCACTTCAGCCTGACCGCCGACAGAATCGACGACG
ACATTCTGAAAGTGATCGTGGACAGCATGAACCACCACGGCGACGCCAGAAGCAAGCTGAGAGAG
GAACTGGCCGAGCTGACCGCTGAGCTGAAGATCTACTCTGTGATCCAGGCCGAGATCAACAAGCAC
CTGAGCAGCTCCGGCACCATCAACATCCACGACAAGAGCATCAACCTGATGGACAAGAATCTGTAC

GGCTACACCGACGAGGAAATCTTCAAGGCCAGCGCCGAGTACAAGATCCTCGAGAAGATGCCCCAG
ACCACCATCCAGGTGGACGGCAGCGAGAAGAAAATCGTGTCCATCAAGGACTTTCTGGGCTCCGAG
AACAAGCGGACAGGCGCCCTGGGCAACCTGAAGAACAGCTACAGCTACAACAAGGACAACAACGA
GCTGAGCCACTTCGCCACCACCTGTAGCGATAAGAGCAGACCCCTGAAC**TGA**

V3

ATGGATAGAGCCAAACTGCTGCTGCTCCTGTTGCTCCTCCTGCTGCCTCAGGCTCAGGCCGTGATGA
TCAGAGCCTACGAGCAGAACCCTCAGCACTTCATCGAGGACCTGGAAAAAGTGCGCGTGGAACAGC
TGACAGGCCACGGAAGTAGCGTGCTGGAAGAACTGGTGCAGCTGGTCAAGGACAAGAACATCGAC
ATCAGCATTAAGTACGACCCTCGGAAGGACAGCGAGGTGTTCGCCAACAGAGTGATCACCGACGAC
ATCGAGCTGCTGAAGAAGATCCTGGCCTACTTCCTGCCTGAGGACGCCATCCTGAAAGGCGGCCACT
ACGACAATCAGCTGCAGAACGGCATCAAGCGCGTGAAAGAGTTCCTGGAAAGCAGCCCCAACACAC
AGTGGGAGCTGAGAGCCTTCATGGCTGTGATGCACTTCAGCCTGACCGCCGACAGAATCGACGACG
ACATTCTGAAAGTGATCGTGGACAGCATGAACCACCACGGCGACGCCAGAAGCAAGCTGAGAGAG
GAACTGGCCGAGCTGACCGCTGAGCTGAAGATCTACTCTGTGATCCAGGCCGAGATCAACAAGCAC
CTGAGCAGCTCCGGCACCATCAACATCCACGACAAGAGCATCAACCTGATGGACAAGAATCTGTAC
GGCTACACCGACGAGGAAATCTTCAAGGCCAGCGCCGAGTACAAGATCCTCGAGAAGATGCCCCAG
ACCACCATCCAGGTGGACGGCAGCGAGAAGAAAATCGTGTCCATCAAGGACTTTCTGGGCTCCGAG
AACAAGCGGACAGGCGCCCTGGGCAACCTGAAGAACAGCTACAGCTACAACAAGGACAACAACGA
GCTGAGCCACTTCGCCACCACCTGTAGCGATAAGAGCAGACCCCTGTGCTTCAGCTACTACCAGAAG
TACATCGAGGGCAACAAGACCTTCGCCATCATTGCCATCGTGTTCGTGTTTATCCTGACCGCTATCCT
GTTCCTGATGAGCCGGCGGTACAGCCGCGAGAAGCAGAAC**TGA**

V4

ATGGATAGAGCCAAACTGCTGCTGCTCCTGTTGCTCCTCCTGCTGCCTCAGGCTCAGGCCGTGATGA
TCAGAGCCTACGAGCAGAACCCTCAGCACTTCATCGAGGACCTGGAAAAAGTGCGCGTGGAACAGC
TGACAGGCCACGGAAGTAGCGTGCTGGAAGAACTGGTGCAGCTGGTCAAGGACAAGAACATCGAC
ATCAGCATTAAGTACGACCCTCGGAAGGACAGCGAGGTGTTCGCCAACAGAGTGATCACCGACGAC
ATCGAGCTGCTGAAGAAGATCCTGGCCTACTTCCTGCCTGAGGACGCCATCCTGAAAGGCGGCCACT
ACGACAATCAGCTGCAGAACGGCATCAAGCGCGTGAAAGAGTTCCTGGAAAGCAGCCCCAACACAC
AGTGGGAGCTGAGAGCCTTCATGGCTGTGATGCACTTCAGCCTGACCGCCGACAGAATCGACGACG
ACATTCTGAAAGTGATCGTGGACAGCATGAACCACCACGGCGACGCCAGAAGCAAGCTGAGAGAG
GAACTGGCCGAGCTGACCGCTGAGCTGAAGATCTACTCTGTGATCCAGGCCGAGATCAACAAGCAC
CTGAGCAGCTCCGGCACCATCAACATCCACGACAAGAGCATCAACCTGATGGACAAGAATCTGTAC
GGCTACACCGACGAGGAAATCTTCAAGGCCAGCGCCGAGTACAAGATCCTCGAGAAGATGCCCCAG
ACCACCATCCAGGTGGACGGCAGCGAGAAGAAAATCGTGTCCATCAAGGACTTTCTGGGCTCCGAG
AACAAGCGGACAGGCGCCCTGGGCAACCTGAAGAACAGCTACAGCTACAACAAGGACAACAACGA
GCTGAGCCACTTCGCCACCACCTGTAGCGATAAGAGCAGACCCCTGAATGGCGGCGGAAGCGGAG
GATCTGGCGGCGATATTATCAAGCTGCTCAACGAGCAAGTGAACAAAGAGATGAACAGCGCCAACC
TGTACATGAGCATGAGCAGCTGGGCCTACACACACAGCCTTGATGGCGCTGGACTGTTCCTGTTTGA
CCACGCCGCCGAGGAATACGAGCACGCCAAGAAGCTGATCATCTTCCTGAACGAGAACAACGTGCC
CGTGCAGCTGACCAGCATTTCTGCCCCTGAGCACAAGTTCGAGGGCCTGACACAGATCTTCCAGAAG
GCCTACGAACACGAGCAGCACATCAGCGAGTCCATCAACAACATCGTGGACCACGCCATTAAGAGC
AAGGATCACGCCACCTTCAACTTTCTGCAGTGGTACGTGGCCGAACAGCACGAGGAAGAGGTGCTG

TTTAAGGACATCCTGGACAAGATCGAGCTGATCGGCAACGAGAACCACGGCCTGTATCTGGCCGAC
CAGTACGTGAAGGGAATCGCCAAGAGCCGGAAGTCC**TGA**
